# Effects of gender, age, experience, and practice on driver reaction and acceptance of traffic jam chauffeur systems

**DOI:** 10.1038/s41598-021-97374-5

**Published:** 2021-09-09

**Authors:** Husam Muslim, Makoto Itoh, Cho Kiu Liang, Jacobo Antona-Makoshi, Nobuyuki Uchida

**Affiliations:** 1 Japan Automobile Research Institution, 2530 Karima, Tsukuba, Ibaraki 305-0822 Japan; 2 Faculty of Engineering, Information and Systems, University of Tsukuba, 1-1-1 Tennoudai, Tsukuba, Ibaraki 305-8573 Japan; 3 Graduate School of Systems and Information Engineering, University of Tsukuba, 1-1-1 Tennoudai, Tsukuba, Ibaraki 305-8573 Japan

**Keywords:** Electrical and electronic engineering, Risk factors

## Abstract

This study conducted a driving simulation experiment to compare four automated driving systems (ADS) designs during lane change demanding traffic situations on highways while accounting for the drivers’ gender, age, experience, and practice. A lane-change maneuver was required when the automated vehicle approaches traffic congestion on the left-hand lane. ADS-1 can only reduce the speed to synchronize with the congestion. ADS-2 reduces the speed and issues an optional request to intervene, advising the driver to change lanes manually. ADS-3 offers to overtake the congestion autonomously if the driver approves it. ADS-4 overtakes the congestion autonomously without the driver’s approval. Results of drivers’ reaction, acceptance, and trust indicated that differences between ADS designs increase when considering the combined effect of drivers’ demographic factors more than the individual effect of each factor. However, the more ADS seems to have driver-like capacities, the more impact of demographic factors is expected. While preliminary, these findings may help us understand how ADS users’ behavior can differ based on the interaction between human demographic factors and system design.

## Introduction

The last two decades have witnessed rapid developments in automated driving technology. All aim to realize an old human vision of self-driving vehicles. The Society of Automotive Engineers categorized this vision into six levels of driving automation escalating from no driving automation to full driving automation^1^. Partial driving automation, which combines the features of lane-keeping assistance and adaptive cruise control systems, represents a borderline between conventional (human-controlled) vehicles and automated vehicles. Partial driving offers a shared responsibility of the dynamic driving task (DDT), which is divided into sustained lateral and longitudinal vehicle motion control (LVMC) that is performed by the system and objects and events detection and response (OEDR) that must be carried out by the driver^2^. Ultimately, when the partial driving system is engaged, the driver is driving, and thus he/she is required to monitor both the system and the roadway, respond appropriately, and retake the vehicle control where needed^3^. Concerns have been expressed on the potential effects of monotonous and predictable driving on drivers’ attention while supervising partial driving systems^4,5^. Such effects may impair drivers’ ability to interact appropriately with partial driving automation and perform the OEDR subtasks^6–8^.

With conditional driving automation mastering the LVMC and OEDR subtasks, the driver is no longer required to monitor the driving environment^1^. However, from the safety perspective, the driver should take control of the vehicle back from the system when necessary or, occasionally, requested by the system. The system’s request to intervene has motivated a large amount of research to understand the effects of drivers’ engagement on their takeover performance^9^. On the one hand, a considerable number of studies have highlighted the undesirable effects of drivers being out-of-the-loop on their ability to perform cognitive processing and retrieve manual control after automated driving^10,11^. On the other hand, some studies show that differences in drivers’ takeover performance when monitoring the roadway or engaged in non-driving-related tasks exist but are not significant^12,13^. When encountering a situation that requires driver intervention, is driver’s engagement during automated driving all that matters?

In the aviation domain, automation is complex, and the pilots, usually a pilot and a copilot, must monitor a high number of parameters^14^. Although maintaining safety in such a complex system is an organizational effort, pilots are highly competent and trained to cope with the dynamically changing workload and situations^14^. The human–machine interface (HMI) in automotive automation could be less complex, but the driving environment is faster-paced and more complex than aviation, and drivers are less qualified than pilots^15^. In both aviation and automotive domains, the performance of the operation and tactical tasks and strategic decisions is highly dependent on humans’ ability to learn from heuristics and experience. These suggest a potential role of age and experience factors when operating airplanes and vehicles and deserve attention on these factors’ influence on control transition between humans and automated systems^16,17^.

Age has been found to affect humans’ hazard perception, reaction time, cognitive processing speed and quality, and task switching ability^18,19^. With manual driving, the driving performance of elderly and experienced drivers when exposed to secondary tasks is less affected than that of younger drivers who performed secondary tasks better with less attention to the driving task^20^. With automated driving, research has found that the takeover (the transferring of vehicle control from ADS to the driver) time by younger drivers was generally shorter than that of older drivers^21,22^. Further surveys have also highlighted the effects of drivers’ age on their acceptance of automated driving vehicles^23^. However, some studies have investigated the association between drivers’ age and gender and found, for example, a significant difference between younger male drivers and older female drivers in terms of reaction time and task performance during different conditions of manual driving^19^.

Recently investigators have examined the effects of training and practice on driver takeover during automated driving^24–28^. These studies established that prior familiarization and practice of automated driving affect drivers’ performance, acceptance, and trust compared to drivers presented with automated driving for the first time. Although the effects of driver demographic factors (e.g., gender, age, experience, and practice) on driver performance, acceptance, and trust have been investigated^29^, the effects of the interaction between these factors and ADS designs on driver’s takeover decision and performance remains unclear. This study attempts to address this gap by evaluating drivers’ interaction with different ADS designs during non-critical automated driving while accounting for driver gender, age, experience, and practice factors.

This paper investigates the impact of human demographic factors on driver decision-making and control when exposed to different ADS designs and traffic conditions. The proposed ADS represents an idealized conditional driving system that can perfectly master the LVMC subtask at low speeds of up to 60 km/h and carry out the OEDR subtasks to a limited extent. The investigated scenarios replicate a conditionally automated vehicle approaching a traffic congestion (20 km/h) while the adjacent lane was available with light traffic circulating at 60 km/h. All test scenarios were not safety critical (no imminent crash), so the main focus could be understanding the accuracy and the promptness of the cognitive processing required to maintain safety during reactive control driving. It was hypothesized that the more the system requires drivers’ decisions and control, the less the drivers accept and trust the system. It was also hypothesized that the drivers would rather use the automated driving functions of the system when available than intervening in the automated process of the system. Finally, we anticipated that the combined effect of driver gender, age, experience, and practice would be more than the individual impact of each factor.

## Method

### Participants and apparatus

Forty volunteer drivers (Female = 20, Male = 20; Age_min_ = 22; Age_max_ = 69; Age_mean_ = 44.5; Age_stdev_ = 15.4) holding a valid driver license participated in a driving simulation experiment. The experiment was approved by the ethical committee of the Faculty of Engineering, Information, and Systems at the University of Tsukuba, Japan. The experimental settings and design were performed in accordance with relevant guidelines and regulations published by the Japanese psychological association (https://psych.or.jp/). All participants signed informed consent and agreed to be a part of this experiment.

**Figure 1:**
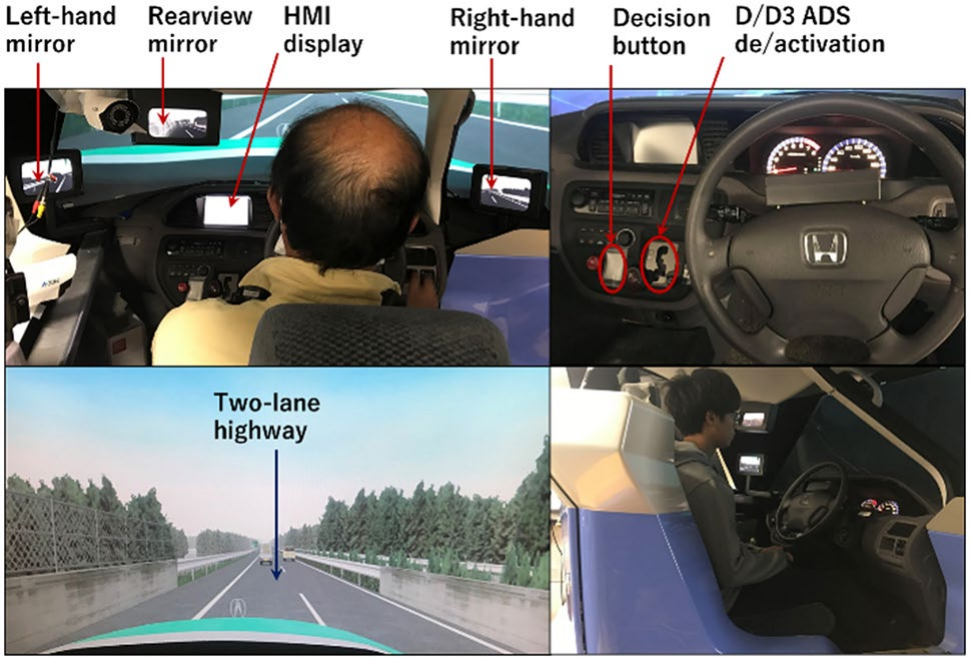
Driving simulator (Honda, Model: DA-1105). Top-left: the driver’s scene (side and rearview mirrors, HMI display, and driver monitoring). Top-right: the simulator interiors (steering wheel, automatic transmission, and dashboard). Bottom-left: the driving environment (front screen). Bottom-right: a participant is sitting inside the cockpit during automated driving.

The experiment was implemented in a medium-fidelity driving simulator built by Honda (Fig. 1). The simulator consists of a dynamic car mockup mounted on four movable legs, in which an actual car seat and dashboard are placed with 120° projection screen and three small LCDs to simulate the front, rear, and side driving views, respectively. The simulator was equipped with conditional driving automation systems with a human–machine interface (HMI) to display the system state and roadway.

An ADS was available at a speed range between zero and 60 km/h based on traffic conditions. The drivers could activate and deactivate the system by shifting the gear stick between D for manual driving and D3 for the automated driving mode. The system had four different states, as described in Table 1. These states were displayed in a separated LCD located in the middle of the dashboard (Fig. 1, right). The toggling of the system status has been associated with an acoustic alert to arouse the driver’s attention. When the system is activated, DDT (i.e., LVMC and OEDR subtasks) can entirely be delegated to the system, such that the driver’s control and monitoring are no longer required.

**Table 1:** HMI display of the automated driving system states. The HMI design was developed by Muslim et al.^13^

System status	Description	HMI
Deactivated	**HMI-1:** The driver performs DDT entirely	
Activated at full speed 60 km/h	**HMI-2:** ADS performs DDT entirely. Driver monitoring of ADS and traffic is optional	
Activated during the traffic jam (< 20 km/h)	**HMI-3:** ADS synchronizes with the traffic jam ahead	
Activated with action required	**HMI-4:** ADS communicates with the driver to overtake the traffic jam	

All test scenarios were conducted on a two-lane highway during the daytime. In the first five minutes of each scenario, the traffic was smooth and light such that the ADS could constantly control the vehicle at full speed (60 km/h). In the sixth minute, the system encountered traffic congestion on the left-hand lane. However, the traffic on the right-hand lane was still smooth, with few cars passing at 60 km/h. The ADS changed its state from HMI-2 (autopilot on) to HMI-3 (autopilot on/slow traffic) and reduced the speed to 20 km/h to synchronize with the slow traffic ahead. The system displays HM-4 (autopilot on/action required) to inform the driver about necessary lane-change maneuvers. However, the system may not return to HMI-1 (autopilot off) unless the driver shifts the gear stick to D3 or overrule the system operation by steering the vehicle or pressing the pedals.

### ADS designs

The autopilot proposed in this study is a limited-speed traffic jam chauffeur (i.e., conditional automated driving) that can perform LVMC and OEDR subtasks for an extended time without driver intervention. It is different from the partially automated systems (e.g., Tesla’s autopilot) in which driver’s monitoring is necessary^30^. When the automated vehicle approached a traffic jam on its main lane and the system displayed HMI-3 (Table 1), changing lanes was recommended to avoid slow traffic and recover the original speed. However, the system’s ability to detect and perform lane change maneuvers varied as follows:

1. ADS-1 (baseline): the system could only keep the lane and continue automated driving at a slow speed (20 km/h). The driver could decide the next course of action whether to take over and change lanes manually or keep the automated driving at a slow speed on the left-hand lane.
2. ADS-2: the system displayed HMI-4 requesting the driver to take over the vehicle control and change lanes. However, the driver could respond to the system’s request to intervene or ignore the request and let the system continue automated driving at 20 km/h.
3. ADS-3: the system displayed HMI-4 requesting the driver’s permission to execute the lane-change maneuver automatically. The driver could approve the automatic lane change by pushing a button (Fig. 1, top-right) or ignore the request and let the system continue automated driving at 20 km/h.
4. ADS-4: the system displayed HMI-4 informing the driver that an automatic lane-change maneuver will start in 6 s. The driver could disapprove of the lane change execution by pushing a button (Fig. 1, right) within the 6 s period; otherwise, the system proceeded with the maneuver.

### Experimental design and procedures

This experiment followed a within-subject repeated measures design such that each driver experienced the four ADS designs. For all participants, the experiment started with a demographic survey (5 min), a brief explanation (15 min), two training drives (5 min each), followed by four testing drives (8–10 min each), and ended with questionnaires. The familiarization and training phase started with a manual drive preceding an automated drive to introduce the participants to the driving simulator and automated driving. Each ADS design was tested once during the testing phase. The order in which the participants encountered the four ADS designs was randomized using the Latin-square method to reduce the experience effects.

The participants were divided into demographic groups and subgroups to investigate the effects of demographic factors (gender, age, experience, and practice) on driver behavior toward the system. First, the participants were categorized based on their gender, i.e., 20 males and 20 females. Each category was divided into two groups (10 drivers each) based on the drivers’ age and driving experience (years of holding a valid driver’s license). The younger group consisted of drivers younger than 45 year-old with driving experience between 1 and 24 years, and the older group consisted of drivers older than 45 year-old with driving experience of more than 25 years. Each age group was further subdivided into two subgroups (5 drivers each) based on participants’ previous practice of automated driving within three months before the current driving experiment. At the end of all testing trials, the participants had to complete post-experiment questionnaires regarding their acceptance of and trust in each ADS design. The participants were asked to mark their answers on a 10 cm line ranged between zero (not at all) and ten (absolutely).

## Results

Table 2 presents descriptive statistics of the type of first drivers’ response to the change in traffic condition and system status. The purpose of the table is to understand the combined effects of the driver’s demographic factors (gender, age, experience, and practice) and the system design implications on the first driver’s control input.

**Table 2:** Statistical data of the first control input by the drivers when encountering the traffic congestion for each system in consideration of drivers’ gender, age, experience, and practice.

Driver demographic factors			The first driver reaction										Lane change maneuvers	
Gender	Age and driving experience	Practice	Hands-on the steering wheel				Decision Button		None					
			*ADS-1*	*ADS-2*	*ADS-3*	*ADS-4*	*ADS-3*	*ADS-4*	*ADS-1*	*ADS-2*	*ADS-3*	*ADS-4*	*Manual*	*Automatic*
Male	Younger	Yes*	5/5	5/5	0/5	0/5	5/5	2/5	0/5	0/5	0/5	3/5	11/20	8/20
		No*	3/5	4/5	0/5	1/5	4/5	3/5	2/5	1/5	1/5	1/5	11/20	5/20
	Older	Yes	5/5	4/5	0/5	1/5	5/5	0/5	0/5	1/5	0/5	4/5	10/20	9/20
		No	4/5	5/5	1/5	2/5	4/5	0/5	1/5	0/5	0/5	3/5	12/20	7/20
Female	Younger	Yes	4/5	5/5	0/5	1/5	5/5	0/5	1/5	0/5	0/5	4/5	10/20	9/20
		No	5/5	4/5	0/5	0/5	5/5	2/5	0/5	1/5	0/5	3/5	11/20	8/20
	Older	Yes	4/5	5/5	1/5	1/5	3/5	2/5	1/5	0/5	1/5	2/5	11/20	5/20
		No	4/5	5/5	0/5	0/5	5/5	0/5	1/5	0/5	0/5	5/5	9/20	10/20
Total			34/40	37/40	2/40	6/40	36/40	9/40	6/40	3/40	2/40	25/40		

*Yes: drivers previously practiced automated driving; No: drivers presented with automated driving for the first time.

It is apparent from the table that the vast majority of the participants (92%) decided to change lanes, manually or automatically, to avoid traffic congestion. Very few participants (8%) decided to keep the lane and continue with automated driving at a slow speed (20 km/h). Although ADS-1 did not support drivers’ decisions or actions when encountering traffic congestion, approximately 85% of the participants took over the vehicle control and manually changed lanes. For ADS-2, 92% of the participants responded to the system’s optional request to resume manual control and overtake the traffic congestion. While both ADS-3 and ADS-4 were able to perform lane change automatically, 90% of the participants pushed the decision button to permit ADS-3 automatic lane-change maneuver, approximately 40% of the participants pushed the decision button to interrupt ADS-4 automatic lane-change maneuver. In general, there was no significant effect of the demographic factors on drivers’ first reaction and choice. However, the design of the system and HMI strategies affected driver’s behavior toward each system more compared to demographic factors.

**Figure 2:**
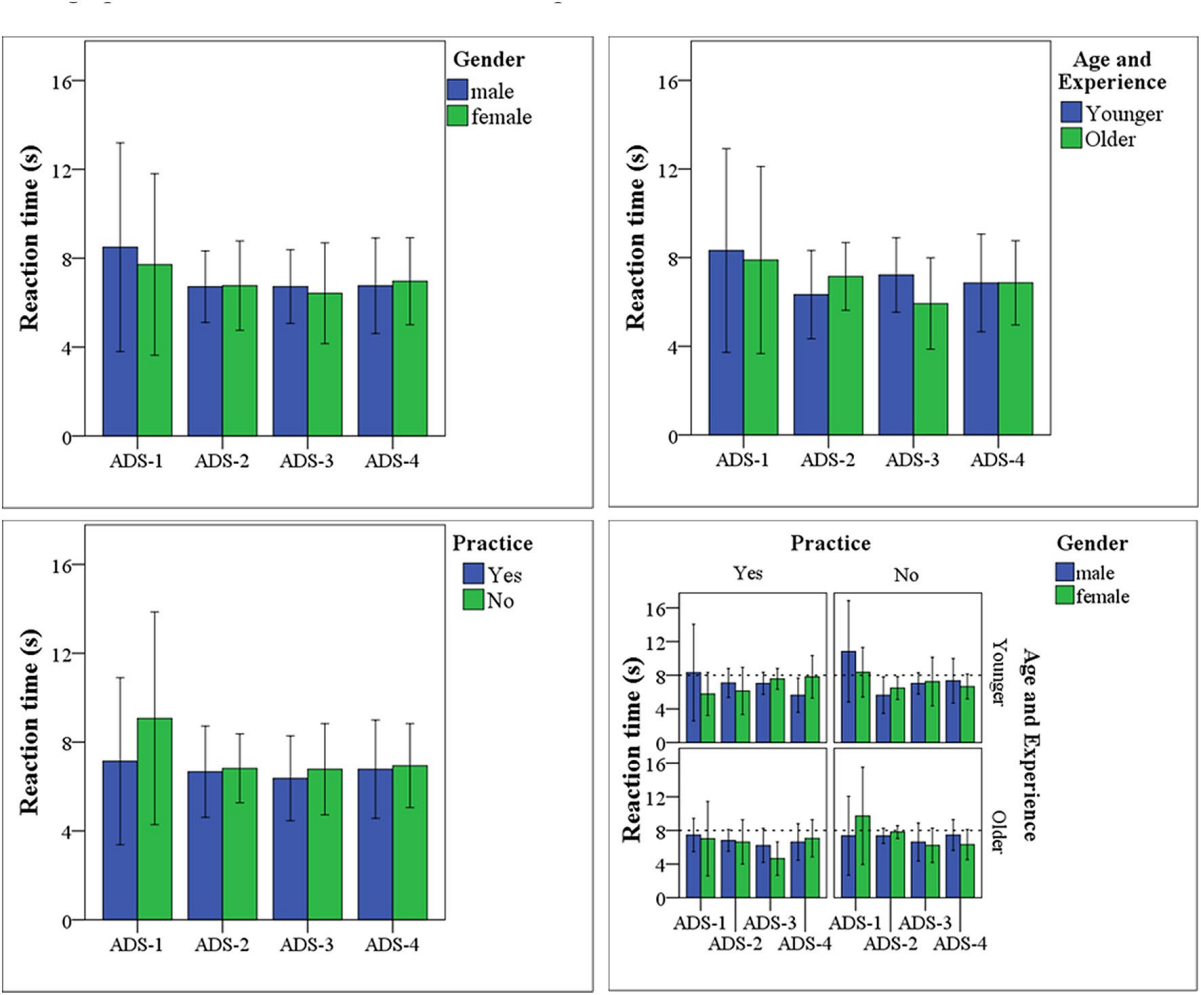
Drivers reaction time in response to the HMI and traffic changes. Top-left: participants are divided into 20 male and 20 female drivers. Top-right: participants are divided into 20 older and 20 younger drivers. Bottom-left: participants are divided into 20 practiced and 20 non-practiced drivers based on their previous experience with automated driving. Bottom-right: participants are divided into eight subgroups (5 drivers each) based on gender, age and experience, and previous practice.

Figure 2 compares drivers’ reaction time among groups and subgroups under each system. The driver reaction time was calculated as the time elapsed from the system triggered HMI-3 to the driver’s first reaction (hands on the wheel, foot on the pedal, or push the decision button) in response to the change in traffic condition. Statistically significant effects were identified for ADS design, gender, age and driving experience as well as practice (F (3, 156) = 4.32, F (1, 38) = 32.46, F (1, 38) = 27.03, F (1, 38) = 36.10 respectively, *p* < 0.01). The analysis also indicate significant interactions occurred between ADS design and gender groups (F (3, 144) = 7.33, *p* < 0.05), ADS design and age and driving experience groups (F (3, 144) = 9.18, *p* < 0.01), and ADS design and previous practice (F (3, 144) = 11.12, *p* < 0.01). In general, the driver’s reaction time results were comparable between the ADS-2 and ADS-3 conditions, but both were shorter than the ADS-1 and ADS-4 conditions. Supporting driver’s decision-making under ADS-2 and ADS-3 might reduce the time spent by the drivers to understand the ADS behavior and traffic condition, which could improve driver’s risk field and perceived risk^31^.

For each demographic factor, multiple comparisons with Tukey HSD indicated that drivers’ reaction time was comparable. Overall, the highest mean level was recorded under the ADS-1 condition (M = 10.83) by the younger male drivers with a first-time practice of automated driving, while the minor mean level was recorded under the ADS-2 condition (M = 4.66) by the older female drivers with the previous practice of automated driving. For ADS-1, the practiced younger female drivers reacted faster than non-practiced younger male drivers (*p* < 0.05). For ADS-2, the non-practiced younger male drivers reacted faster than the non-practiced older female drivers (*p* < 0.05). The analysis indicated a significant difference between practiced younger female drivers and older female drivers under the ADS-3 condition (*p* < 0.05) and between the practiced younger males and females (*p* < 0.01) under the ADS-4 condition. These results indicate that while the effects of driver demographic factors may not be significant when considered separately, the combined effects of drivers’ gender, age, experience, and practice are more noticeable. They are consistent with our anticipation that the combined effect of driver demographic factors would be more than the separated effect of each factor.

**Figure 3:**
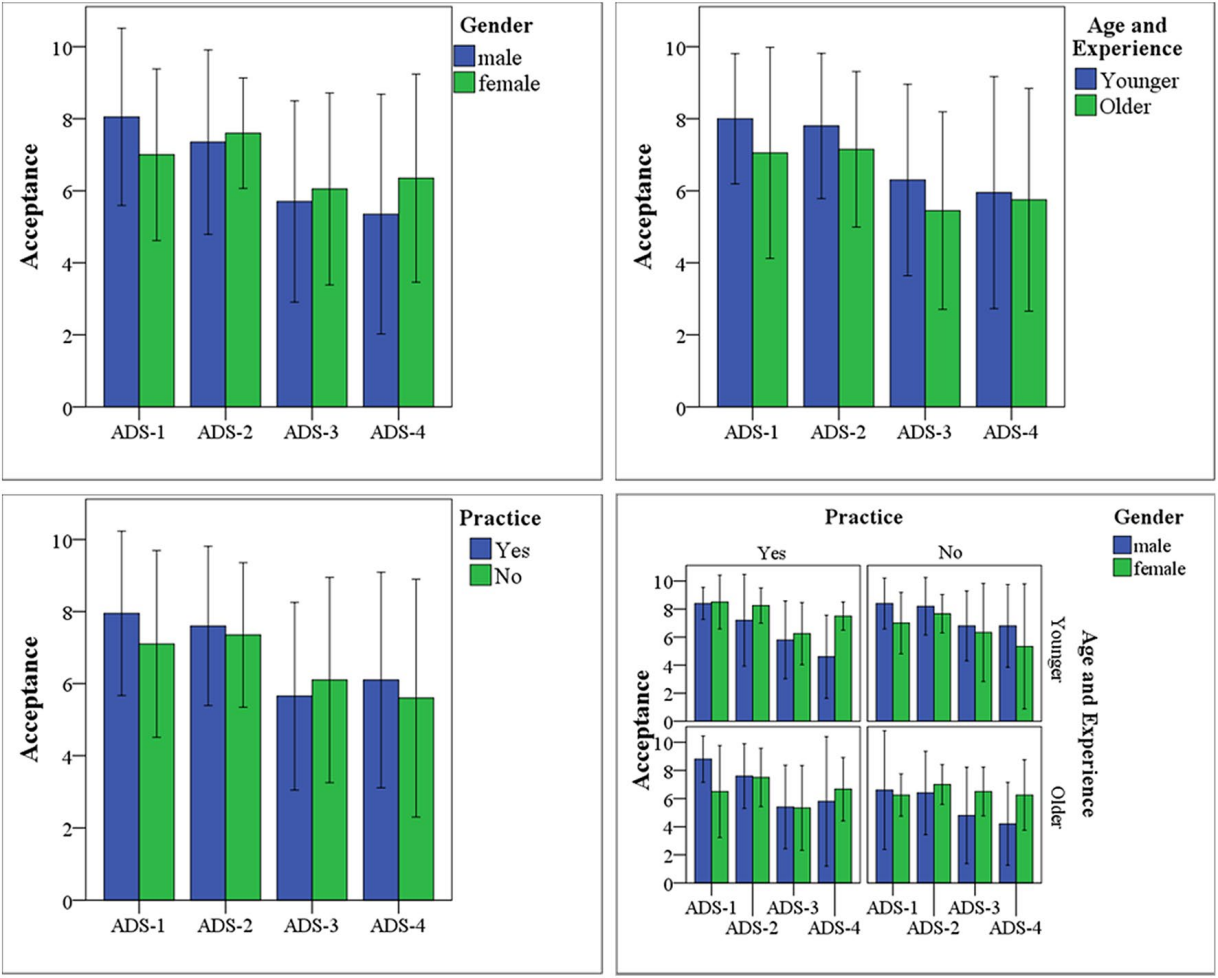
Subjective assessment of drivers’ acceptance of ADS considers participants’ gender, age, experience, and practice.

Drivers’ acceptance of each ADS was evaluated based on their willingness to use the system in the real world. The question was administrated to the participants after completing all driving tests. Figure 3 compares drivers’ rating of their acceptance of each ADS design between groups and subgroups. In general, ADS-1 and ADS-2 were more accepted than ADS-3 and ADS-4. These results support the first hypothesis that the more the system requires drivers’ decisions and control, the less the drivers accept the system. Each demographic factor resulted in differences between groups for each ADS design. These differences became significant when all factors, i.e., gender, age, experience, and practice, are considered collectively. The practiced older male drivers recorded the highest acceptance rate under the ADS-1 condition, and the non-practiced older male drivers recorded the lowest acceptance rate under the ADS-4 condition.

Wilcoxon Rank Sum test was applied to examine the differences between groups and subgroups within and between systems. The practiced older male drivers significantly more accepted ADS-1 than the non-practiced older female drivers (Z =  − 2.03, *p* < 0.05). However, comparisons of the acceptance of ADS-2 and ADS-3 did not reveal any significant difference between groups and subgroups. ADS-4 was significantly less accepted by the practiced younger male drivers and non-practiced older male drivers than the practiced younger female drivers (Z =  − 3.57, *p* < 0.01) and practiced older female drivers (Z =  − 2.89, *p* < 0.05) respectively.

**Figure 4:**
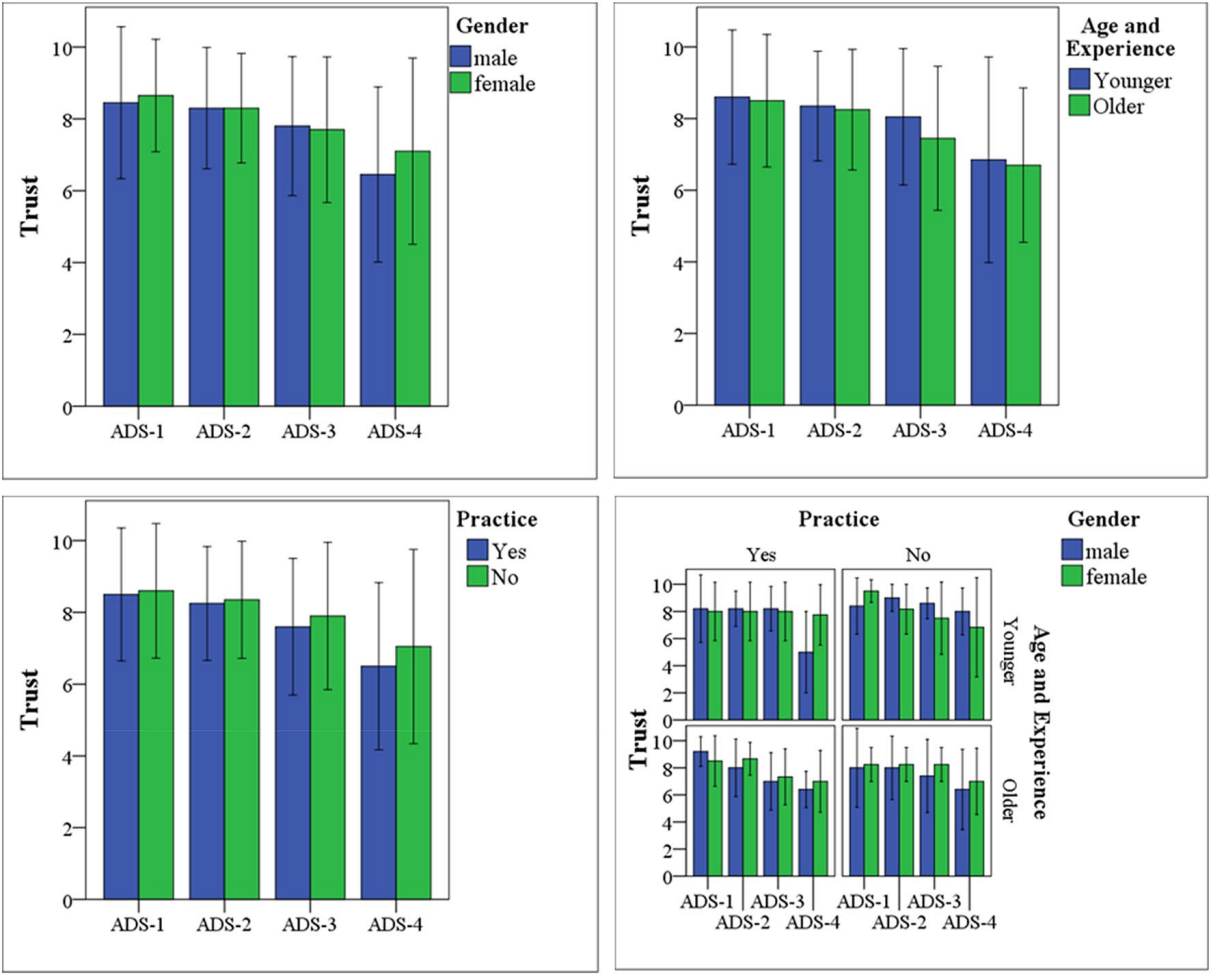
Subjective assessment of drivers’ trust in ADS considers participants’ gender, age, experience, and practice.

The subjective assessment of the participants’ trust in the system was collected under each ADS design, as shown in Fig. 4. The question was to what extent they think the system is trustworthy. Although the participants’ rating of all systems was above the mid-value of the scale, ADS-4 was rated lower than other systems. When the participants were asked about the reason, they reported that it was difficult to trust a system that gives a short time (6 s) to decide whether they have to cancel its action or not in the presence of other vehicles passing at a higher speed on the adjacent lane.

The data in Fig. 4 shows that while the non-practiced younger female drivers recorded the highest trust rate under ADS-1, the practiced younger male drivers recorded the lowest trust rate under ADS-4. Wilcoxon Rank Sum test indicated that the practiced younger female drivers significantly more trusted ADS-4 than the practiced younger male drivers (Z =  − 1.99, *p* < 0.05). However, comparisons of the trust in ADS-1, ADS-2, and ADS-3 did not reveal any significant difference between groups and subgroups. These results indicate that ADS-1, ADS-2, and ADS-3 are affected by the demographic factors to a lesser extent than ADS-4, in which the system has a higher capability of decision making and action implementation without driver’s intervention. Given that the majority of the drivers preferred to change lanes, the finding of ADS-4 condition is contrary to previous studies which have suggested that the more an automated system seems to have human-like behavior, the more human is expected to trust it^32,33^.

## Discussions

This driving simulator study investigated the effects of drivers’ gender, age, experience, and practice on their decision-making and control during low-speed conditional automated driving on a highway. The interaction between the investigated demographic factors and different ADS designs and capabilities resulted in a significant difference in drivers’ decision-making, reaction time, acceptance, and trust. When the drivers encountered traffic congestions during automated driving, they preferred to change lanes more than continue automated driving in the slow lane. Depending on ADS capabilities, the drivers resumed the vehicle control and changed lanes manually or provided an appropriate intervention to let the system changes lanes automatically.

Results of the type of the first control input by the drivers showed that the younger drivers were less likely to interrupt the automated driving compared to the older drivers. There was no significant difference in drivers’ reaction time associated with gender, but drivers who practiced automated driving before reacted faster to the HMI and traffic changes than first-time-practicing drivers. The results showed that the standard deviation of the drivers’ reaction time under the ADS-1 condition is larger than the ADS-2 condition, and the latter is larger than the ADS-3 and ADS-4 conditions. With ADS-1, the drivers had to perceive the traffic change and decide what to do without system support. ADS-2 supports drivers’ decision-making reduced the time spend by the drivers to reach a decision and act^31^. Further reduction in the standard deviation of drivers’ reaction time was achieved when the system supported drivers’ control with a time limitation to decide. These results support the second hypothesis that the drivers would rather use the automated driving functions of the system when available than intervening in the automated process of the system.

The overall drivers’ subjective assessments of their acceptance of and trust in the systems were above the mean. While the human demographic factors revealed significant differences under the ADS-1 and ADS-4 conditions, they did not reveal significant differences under the ADS-2 and ADS-3 conditions. The likely cause for such variance is related to the extent to which the design of each system is compatible with the concept of human-centered automation (see^34,35^ for more details). ADS-1 and ADS-4 did not support human decision-making when the automated process changed in response to an external change in the surrounding environment. It was difficult for the drivers to understand the automated process of ADS-4, which could lead to automation surprises and reduce human trust and acceptance. Although ADS-2 and ADS-3 differed in terms of systems capabilities, both systems are designed to support human decision-making and support drivers’ understanding of the automated process and the surrounding environment. These findings warrant future research on the influence of human and individual characteristics on user-ADS interaction and the design of automated vehicles.

## Conclusions

Although the investigated scenarios were not time-critical, drivers’ decision-making was safety–critical as they had to scan the adjacent lane before deciding to proceed with lane change initiation. The investigation of the interaction between demographic factors and system design in such time-critical conditions has shown that the drivers tend to accept and trust systems with less intervention requirement than systems requiring driver intervention. This study has gone some way toward enhancing our understanding of how driver gender, age, experience, and practice will influence the potential effects of automated vehicles on traffic flow and safety. It also shows that cooperative ADS designs (e.g., ADS-2 and ADS-3) would compromise the influence of demographic factors. Though limited in terms of the small sample size and the investigated scenarios, these findings can be used to develop ADS interventions, particularly during the penetration of automated vehicles in real traffic and the potential conflict with the manually (human) controlled vehicles.

## References

[1] SAE International, Taxonomy and definitions for terms related to driving automation systems for on-road motor vehicles (J3016), ed: Society for Automotive Engineers (2018).

[2] Roe M (2019). Who’s driving that car: An analysis of regulatory and potential liability frameworks for driverless cars. BCL Rev..

[3] Zhou H, Itoh M, Kitazaki S (2020). Effect of instructing system limitations on the intervening behavior of drivers in partial driving automation. Cogn. Technol. Work.

[4] Banks VA, Eriksson A, O’Donoghue J, Stanton NA (2018). Is partially automated driving a bad idea? Observations from an on-road study. Appl. Ergon..

[5] Cabrall CD, Eriksson A, Dreger F, Happee R, de Winter J (2019). How to keep drivers engaged while supervising driving automation? A literature survey and categorisation of six solution areas. Theor. Issues Ergon. Sci..

[6] De Winter JC, Happee R, Martens MH, Stanton NA (2014). Effects of adaptive cruise control and highly automated driving on workload and situation awareness: A review of the empirical evidence. Transport. Res. Part F: Traffic Psychol. Behav..

[7] Kraft A-K, Naujoks F, Wörle J, Neukum A (2018). The impact of an in-vehicle display on glance distribution in partially automated driving in an on-road experiment. Transport. Res. Part F: Traffic Psychol. Behav..

[8] Louw, T., Kountouriotis, G., Carsten, O. & Merat, N. Driver inattention during vehicle automation: How does driver engagement affect resumption of control? in *4th International Conference on Driver Distraction and Inattention (DDI2015), Sydney: Proceedings* (ARRB Group, 2015).

[9] Inagaki T, Sheridan TB (2019). A critique of the SAE conditional driving automation definition, and analyses of options for improvement. Cogn. Technol. Work.

[10] Zeeb K, Buchner A, Schrauf M (2016). Is take-over time all that matters? The impact of visual-cognitive load on driver take-over quality after conditionally automated driving. Accid. Anal. Prev..

[11] Kyriakidis M (2019). A human factors perspective on automated driving. Theor. Issues Ergon. Sci..

[12] Rauffet P, Botzer A, Chauvin C, Saïd F, Tordet C (2020). The relationship between level of engagement in a non-driving task and driver response time when taking control of an automated vehicle. Cogn. Technol. Work.

[13] Muslim, H., Liang, C. K. & Itoh, M. Effectiveness and driver acceptance of sharing decision and control in automated driving, in *2020 IEEE International Conference on Systems, Man, and Cybernetics (SMC)*, 4447–4452 (IEEE, 2020).

[14] Chialastri A (2012). Automation in Aviation.

[15] Cohen J, Nuckolls L, Mourant RR (2006). Endoscopy simulators: Lessons from the aviation and automobile industries. Gastrointest. Endosc. Clin..

[16] Cummings ML, Guerlain S (2007). Developing operator capacity estimates for supervisory control of autonomous vehicles. Hum. Factors.

[17] Barnhart RK, Marshall DM, Shappee E (2020). Introduction to Unmanned Aircraft Systems.

[18] Cantin V, Lavallière M, Simoneau M, Teasdale N (2009). Mental workload when driving in a simulator: Effects of age and driving complexity. Accid. Anal. Prev..

[19] Skrypchuk, L., Mouzakitis, A., Langdon, P. & Clarkson, P. The effect of age and gender on task performance in the automobile, in *Cambridge Workshop on Universal Access and Assistive Technology*, 17–27 (Springer, 2018).

[20] Getzmann S, Arnau S, Karthaus M, Reiser JE, Wascher E (2018). Age-related differences in pro-active driving behavior revealed by EEG measures. Front. Hum. Neurosci..

[21] Körber M, Gold C, Lechner D, Bengler K (2016). The influence of age on the take-over of vehicle control in highly automated driving. Transport. Res. Part F: Traffic Psychol. Behav..

[22] Wu Y, Kihara K, Takeda Y, Sato T, Akamatsu M, Kitazaki S (2019). Effects of scheduled manual driving on drowsiness and response to take over request: A simulator study towards understanding drivers in automated driving. Accid. Anal. Prev..

[23] Huff Jr., E. W., DellaMaria, N., Posadas, B. & Brinkley, J. Am I too old to drive? Opinions of older adults on self-driving vehicles, in *The 21st International ACM SIGACCESS Conference on Computers and Accessibility*, 500–509 (2019)

[24] Zhou H, Kamijo K, Itoh M, Kitazaki S (2021). Effects of explanation-based knowledge regarding system functions and driver’s roles on driver takeover during conditionally automated driving: A test track study. Transport. Res. Part F: Traffic Psychol. Behav..

[25] Ebnali M, Hulme K, Ebnali-Heidari A, Mazloumi A (2019). How does training effect users’ attitudes and skills needed for highly automated driving?. Transport. Res. Part F: Traffic Psychol. Behav..

[26] Hergeth S, Lorenz L, Krems JF (2017). Prior familiarization with takeover requests affects drivers’ takeover performance and automation trust. Hum. Factors.

[27] Forster Y, Hergeth S, Naujoks F, Krems J, Keinath A (2019). User education in automated driving: Owner’s manual and interactive tutorial support mental model formation and human-automation interaction. Information.

[28] Payre W, Cestac J, Delhomme P (2016). Fully automated driving: Impact of trust and practice on manual control recovery. Hum. Factors.

[29] Morales-Alvarez W, Sipele O, Léberon R, Tadjine HH, Olaverri-Monreal C (2020). Automated driving: A literature review of the take over request in conditional automation. Electronics.

[30] Tenhundfeld NL, de Visser EJ, Ries AJ, Finomore VS, Tossell CC (2020). Trust and distrust of automated parking in a Tesla Model X. Hum. Factors.

[31] Kolekar S, de Winter J, Abbink D (2020). Human-like driving behaviour emerges from a risk-based driver model. Nat. Commun..

[32] Waytz A, Heafner J, Epley N (2014). The mind in the machine: Anthropomorphism increases trust in an autonomous vehicle. J. Exp. Soc. Psychol..

[33] Basu, C., Yang, Q., Hungerman, D., Sinahal, M. & Draqan, A. D. Do you want your autonomous car to drive like you? in *2017 12th ACM/IEEE International Conference on Human-Robot Interaction (HRI)*, 417–425 (IEEE, 2017).

[34] Billings CE (2018). Aviation Automation: The Search for a Human-Centered Approach.

[35] Sheridan, T. B. Human centered automation: oxymoron or common sense? in *1995 IEEE International Conference on Systems, Man and Cybernetics. Intelligent Systems for the 21st Century*, Vol. 1, 823–828 (IEEE, 1995).

